# Supplementary material for: Dynamic Coupling of Tyrosine 185 with the Bacteriorhodopsin Photocycle, as Revealed by Chemical Shift Assisted AF-QM/MM Calculations and Molecular Dynamic Simulations
Source: Int J Mol Sci. 2021 Dec 18;22(24):13587. doi: 10.3390/ijms222413587 (PMC8709120; doi:10.3390/ijms222413587)
Supplement: Supplementary file 1 [file ijms-22-13587-s001.zip › ijms-1459026-supplementary.pdf]

Supplementary Materials

Dynamic coupling of tyrosine 185 with the bacteriorhodopsin photocycle, as revealed by chemical shifts, assisted AF-QM/MM calculations and molecular dynamic simulations

Sijin Chen [1,†], Xiaoyan Ding [1,2,†], Chao Sun [1,3], Anthony Watts [2], Xiao He [3,4,*], and Xin Zhao [1,*]

[1] School of Physics and Electronic Science, East China Normal University, 500 Dongchuan Road, Minhang District, Shanghai 200241, P.R. China
[2] Department of Biochemistry, University of Oxford, South Park Road, Oxford OX1 3QU, UK
[3] Shanghai Engineering Research Center of Molecular Therapeutics and New Drug Development, School of Chemistry and Molecular Engineering, East China Normal University, Shanghai, 200062, P.R. China
[4] New York University-East China Normal University Center for Computational Chemistry, NYU Shanghai, Shanghai, 200062, P.R. China
* Correspondence: xzhao@phy.ecnu.edu.cn, xiaohe@phy.ecnu.edu.cn
† These authors contributed equally to this work.

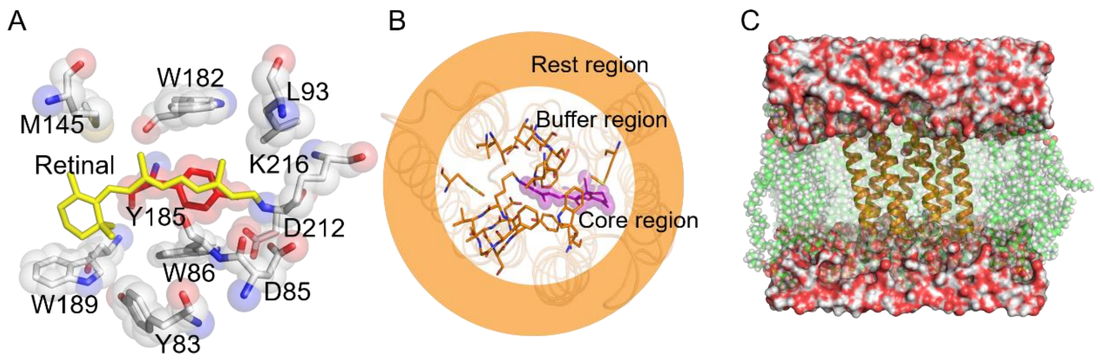

Figure S1. Schematic diagram of the simulation system: (A) relative positions of Y185 and Schiff base in the retinal pocket. The yellow stick model represents the retinal chromophore, Y185 shows in red stick-spheres, and SB and other binding pocket residues are in grey stick-spheres. (B) AF-QM/MM model. The translucent purple and orange sticks represent the core region, other sticks represent the buffer region, and the remaining part is in orange cartoons. (C) Schematic diagram of the MD simulation of bacteriorhodopsin. Orange color represents the bR monomer; oxygen atoms of water molecules are shown as red spheres and surface; POPC lipids are in green spheres; hydrogen atoms are in white.

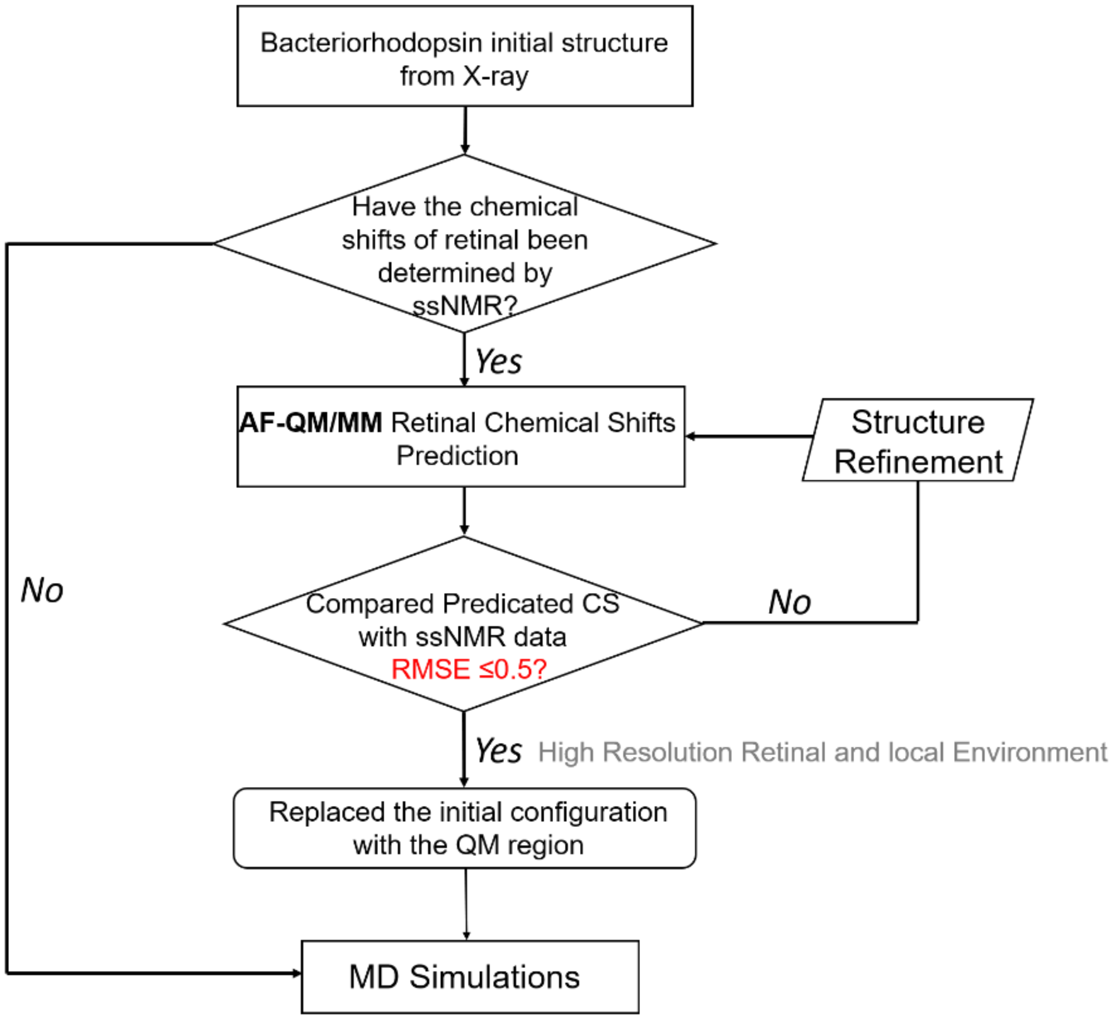

Figure S2. Workflow of the computation algorithm.

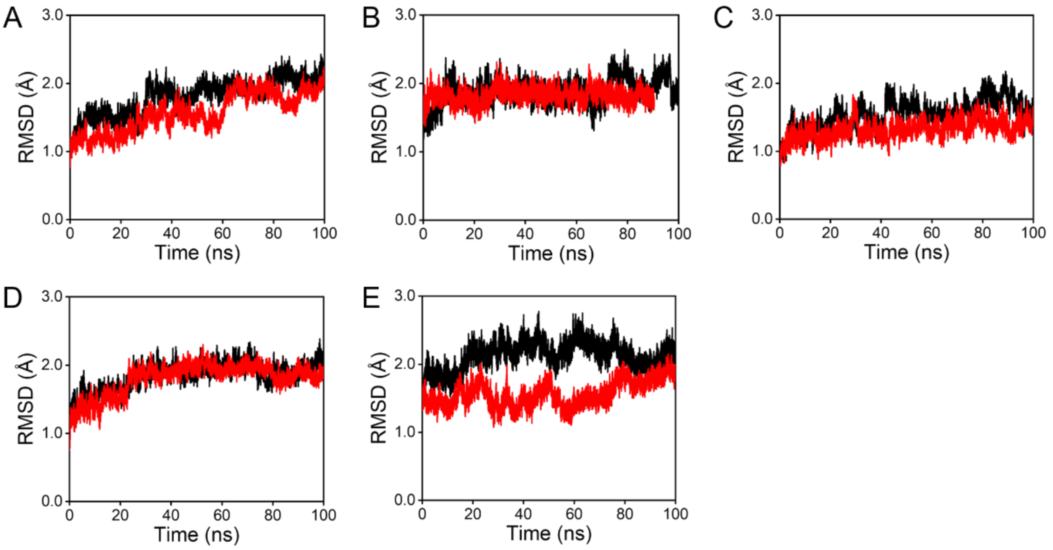

Figure S3. Time evolutions of root mean square deviation (RMSD) of the Cα atoms of WT-bR (black) and Y185F (red) in (A) ground state (light-adapted), (B) M_1, (C) M_2, (D) M_2', and (E) N states.

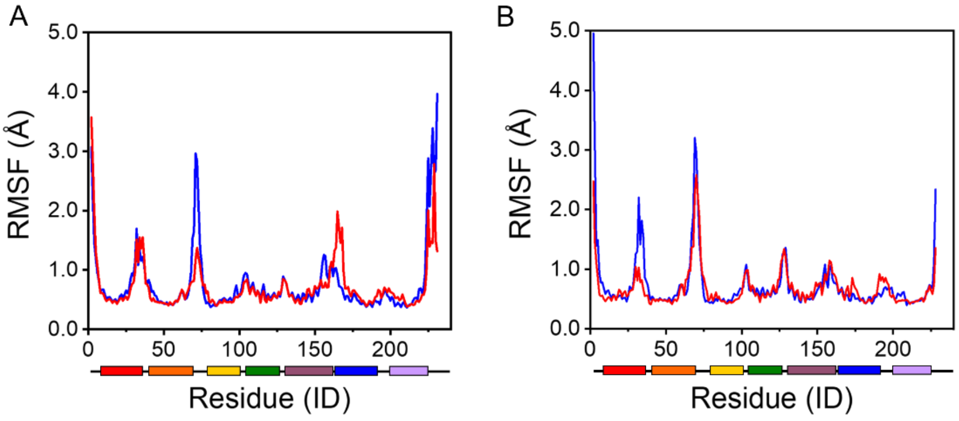

Figure S4. Root mean square fluctuations (RMSF) of the Cα atoms during the simulations in WT-bR (blue) and Y185F (red): (A) WT-bR$_{trans}$ vs. Y185F-bR$_{trans}$; (B) WT-bR$_{cis}$ vs. Y185F-bR$_{cis}$.

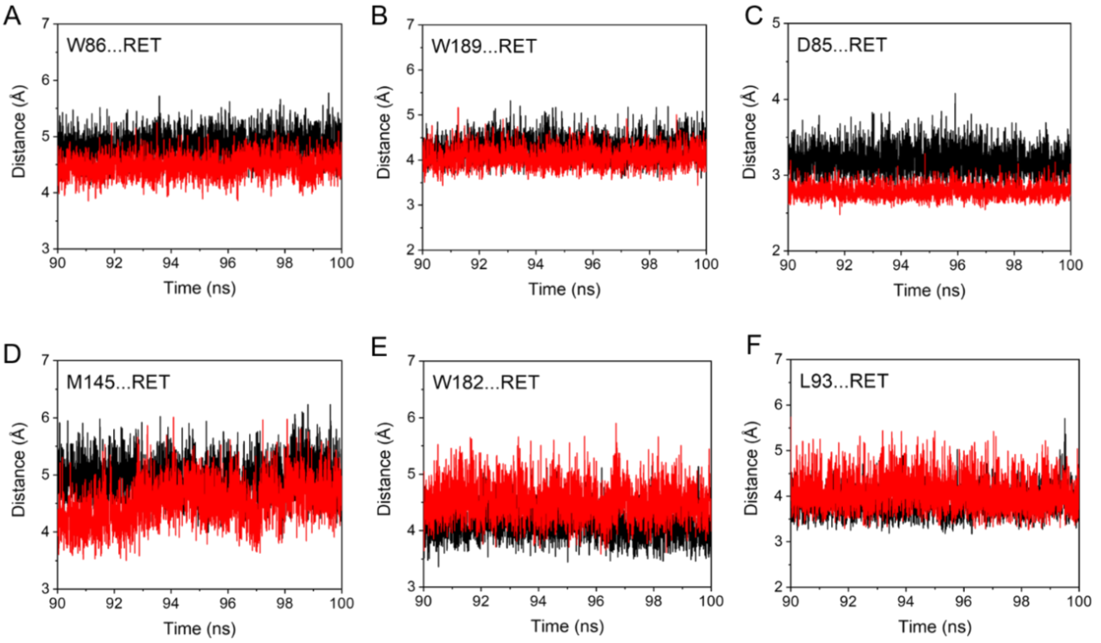

Figure S5. Time evolution of the distance between several key residues and retinal from the last 10 ns simulations of WT-bR*trans* (black) and Y185F-bR*trans* (red).

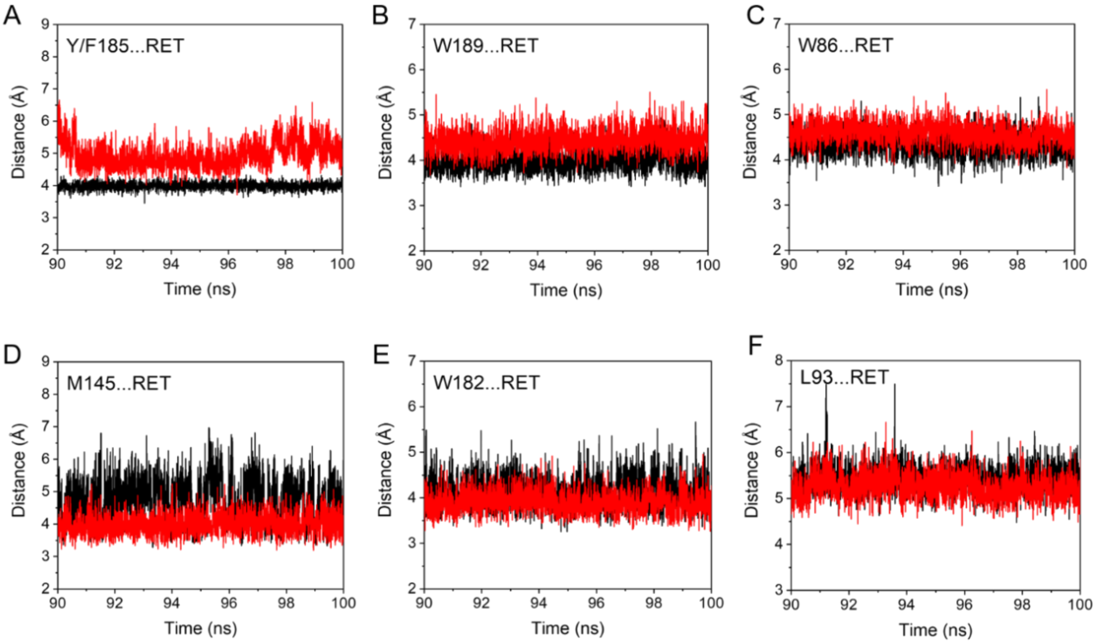

Figure S6. Time evolution of the distance between several key residues and retinal from the last 10 ns simulations of WT-bR*cis* (black) and Y185F-bR*cis* (red).

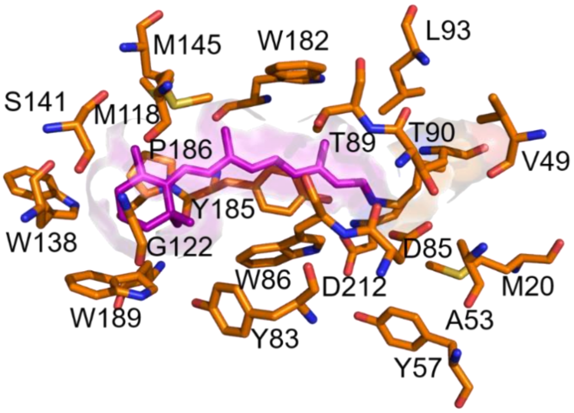

Figure S7. Schematic diagram of the calculated volume size of the retinal binding pocket.

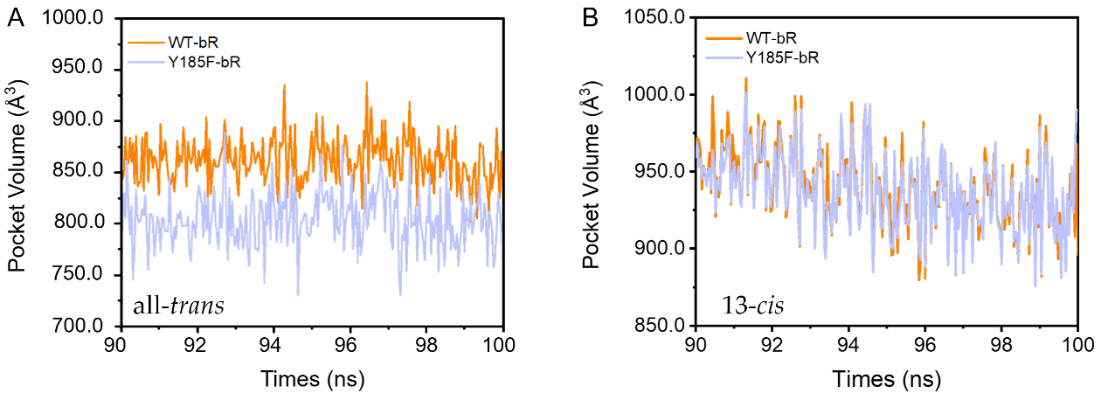

Figure S8. Time evolution of the retinal binding pocket volume in the last 10 ns MD simulations of bR_{trans} and bR_{cis}: (A) WT-bR_{trans} (orange) vs. Y185F-bR_{trans} (light blue), and (B) WT-bR_{cis} (orange) vs. Y185F-bR_{cis} (light blue).

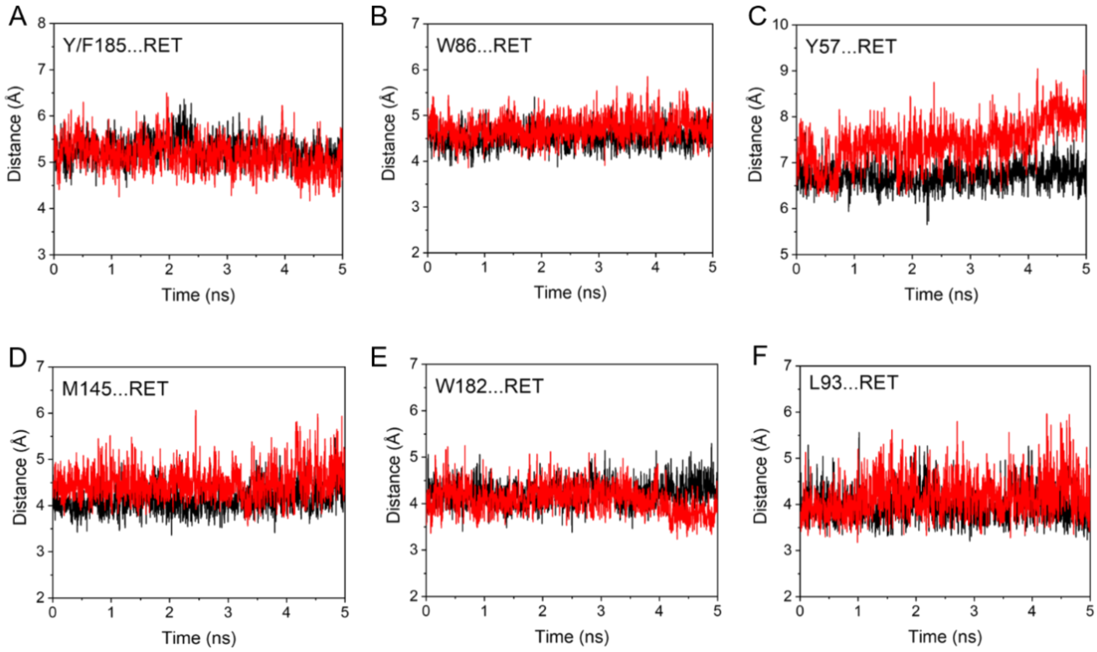

Figure S9. The evolution of distance between the key residues and retinal from the last 5 ns simulations of WT-M_1-es (black) and Y185F-M_1-es (red).

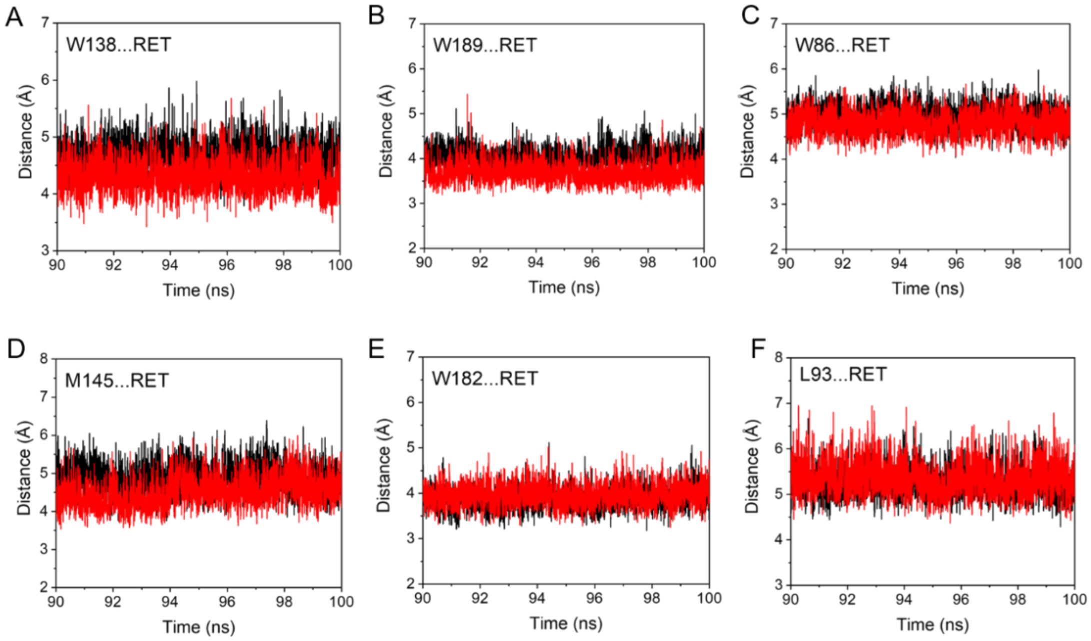

Figure S10. The evolution of distance between the key residues and retinal from the last 10 ns simulations of WT-M_1-cs (black) and Y185F-M_1-cs (red).

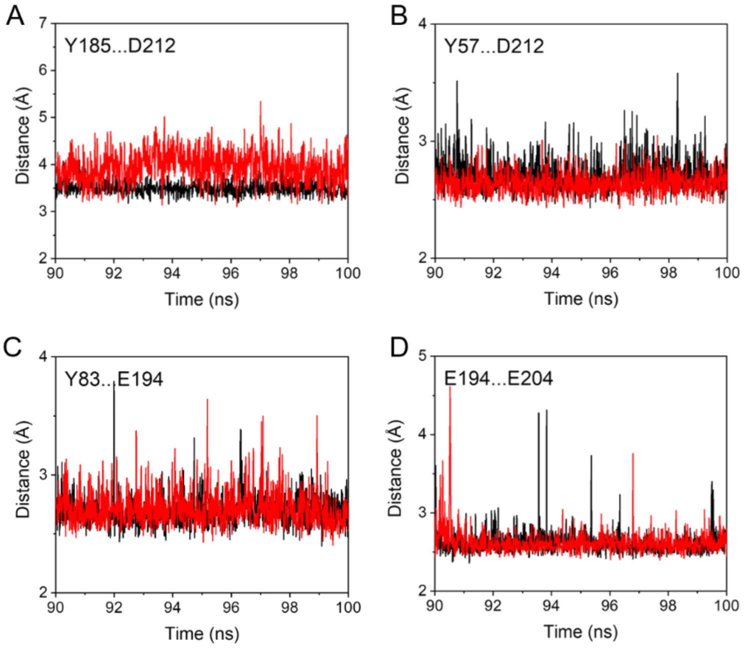

Figure S11. The evolution of distance between the key residues and retinal from the last 10 ns simulations of WT-M_2 (black) and Y185F-M_2 (red).

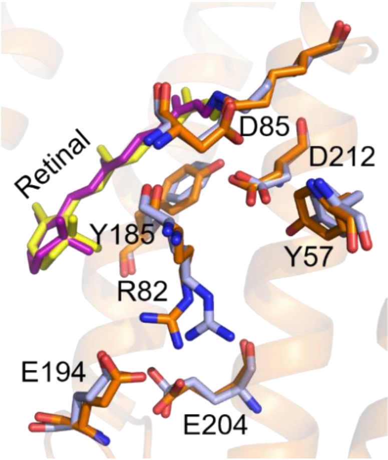

Figure S12. Superposition of the key residues in the extracellular side of WT-M_2 (purple and orange sticks) and Y185F-M_2 (yellow and light blue sticks).

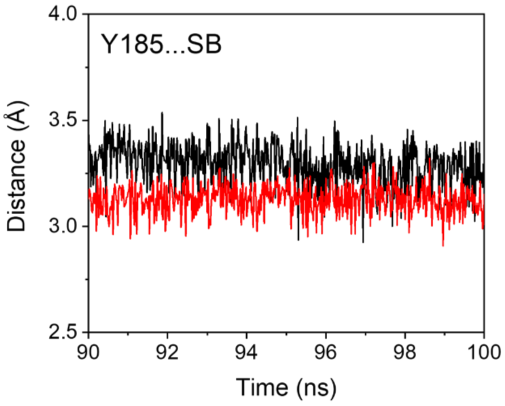

Figure S13. The distance between Y185 and the Schiff base from the repeated simulations of WT-bR_{trans} (black) and WT-bR_{cis} (red).

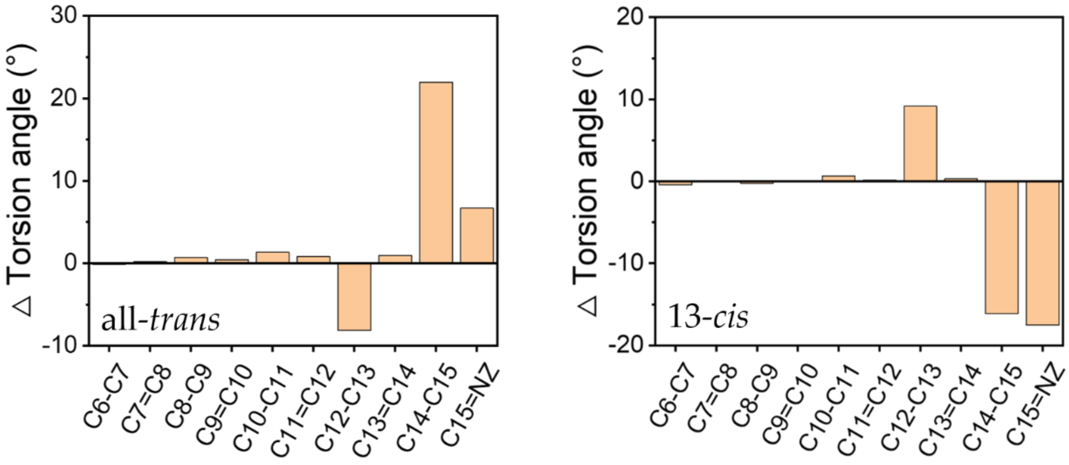

Figure S14. The second time simulation showed the difference in the retinal sidechain torsion angle of Y185F minus WT-bR in the bR_{trans} and bR_{cis}.

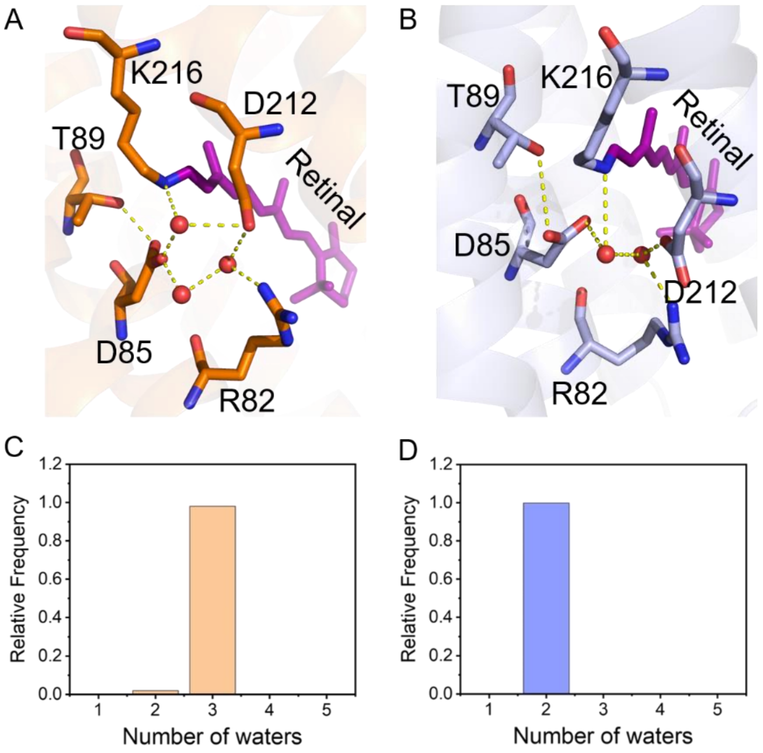

Figure S15. Diagram of the pentagonal H-bond network in WT-bR and Y185F from the repeated simulations: (A) and (B) schematic diagrams of the pentagonal hydrogen bonding network in WT-bR and Y185F-bR from the last snapshot of the simulations. Color codings are the same as used in Figure S12, and red spheres indicate the water molecules. (C) and (D) Occurrence frequency histogram of water molecules within 4.0 Å around D212, D85, R82, and SB in WT-bR (orange) and Y185F-bR (light blue), respectively.

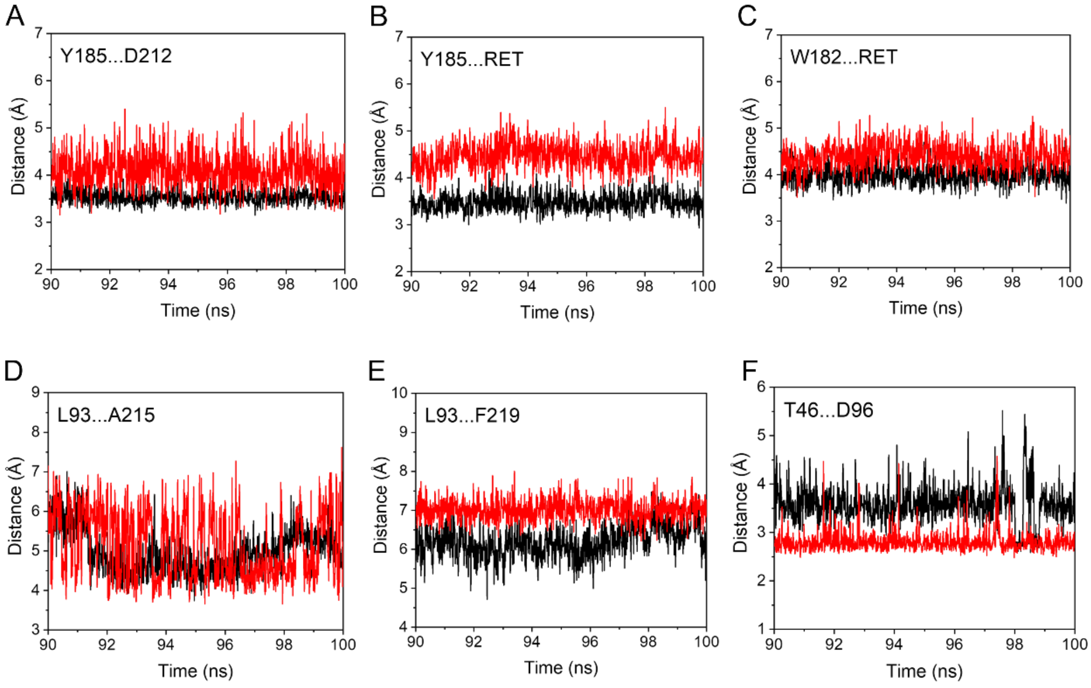

Figure S16. The evolution of distance between the key residues and retinal from the last 10 ns simulations of WT-M2′ (black) and Y185F-M2′ (red).

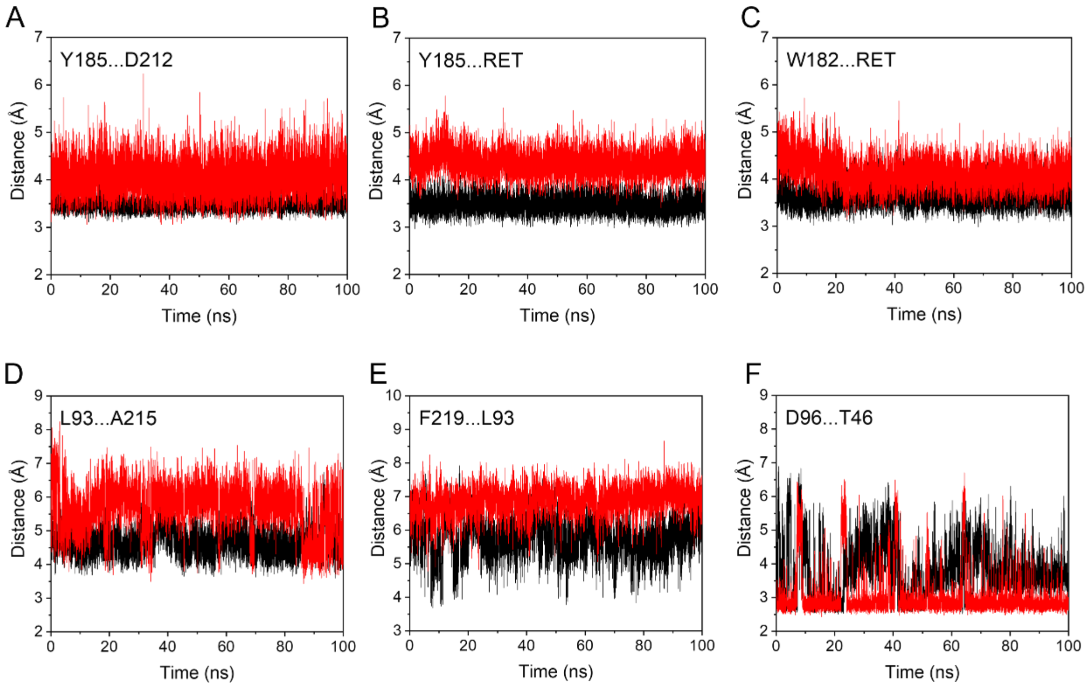

Figure S17. The evolution of distance between the key residues and retinal from the throughout the whole simulations of WT-M2′ (black) and Y185F-M2′ (red).

Table S1. Summary of the MD simulations performed.

Sim	Simulated Model	AF-QM/MM [2]	Length of MD (ns)	States
Sim1	WT-bR$_{trans}$ (1BRR) [1]	Y	100	DA state
Sim2	Y185F-bR$_{trans}$ (1BRR) [1]	Y	100	DA state
Sim3	WT-bR$_{cis}$ (1X0S) [1]	Y	100	DA state
Sim4	Y185F-bR$_{cis}$ (1X0S) [1]	Y	100	DA state
Sim5	WT-M$_1$ (1M0M) [1]	Y	90.36	M$_1$ state
Sim6	Y185F-M$_1$ (1M0M) [1]	Y	100	M$_1$ state
Sim7	WT-M$_2$ (1F4Z) [1]	Y	100	M$_2$ state
Sim8	Y185F-M$_2$ (1F4Z) [1]	Y	100	M$_2$ state
Sim9	WT-M$_2$ (1C8S) [1]		100	M$_2$′ state
Sim10	Y185F-M$_2$ (1C8S) [1]		100	M$_2$′ state
Sim11	WT-bR$_N$ (1C8S) [1]		100	N state
Sim12	Y185F-bR$_N$ (1C8S) [1]		100	N state
Sim13	WT-bR$_{Ptrans}$ (1C3W) [1]		100	LA state
Sim14	Y185F-bR$_{Ptrans}$ (1C3W) [1]		100	LA state

[1] The content in brackets indicates the PDB code of the selected initial crystal structure.
[2] The column indicates the use of AF-QM/MM for refining the retinal before the MD simulations. Y means used.

Table S2. Comparison of the repeated simulation results of the retinal dihedral angles in bR$_{trans}$ and bR$_{cis}$.

Dihedrals/°	C9=C10	C10-C11	C11=C12	C12-C13	C13=C14	C14-C15	C15=N
1_WT-bR$_{trans}$	173.81±4.67	167.95±8.71	174.50±4.16	168.73±8.41	174.25±4.45	151.62±12.80	162.66±8.49
1_Y185F-bR$_{trans}$	174.94±3.91	169.37±8.04	174.97±3.83	159.95±11.14	174.41±4.21	169.49±7.91	169.81±7.02
2_WT-bR$_{trans}$	174.60±4.11	168.03±9.09	173.79±4.60	167.99±8.76	173.09±4.83	147.83±13.28	161.09±8.50
2_Y185F-bR$_{trans}$	175.04±3.74	169.36±7.91	174.65±3.99	159.84±10.92	174.05±4.54	169.81±7.76	167.78±7.58
1_WT-bR$_{cis}$	174.87±3.90	170.07±7.77	175.05±3.72	154.45±11.72	5.70±4.35	171.33±6.31	24.80±10.19
1_Y185F-bR$_{cis}$	174.55±4.09	169.41±8.03	175.11±3.69	167.50±9.13	5.99±4.67	159.20±10.32	9.96±7.61
2_WT-bR$_{cis}$	174.96±3.79	167.92±8.96	174.83±3.77	160.04±13.46	7.59±5.43	172.44±5.76	27.54±10.48
2_Y185F-bR$_{cis}$	175.02±3.82	168.60±8.08	175.00±3.82	169.23±8.00	7.90±5.48	156.31±9.14	10.00±7.25

[1] The number indicates the simulation order.

Table S3. Distance between the key residues and retinal in the last 10 ns simulations of WT-bR$_{trans}$.

Distance/Å	D85-T89	D212-Y185	D212-Y57	R82-D212	D96-T46	W182-C20 [3]	L93-C20 [3]
WT-bR$_{trans}$	2.69±0.12	2.62±0.09	2.66±0.11	2.74±0. 08	2.98±0.28	4.10±0.26	3.86±0.29
1BRR [1]	2.7	2.8	2.7	4.1	2.7	4.0	3.8
1C3W [2]	2.8	2.6	2.5	3.8	2.6	4.3	3.9

[1] Essen, L., et al., *Proceedings of the National Academy of Sciences of the United States of America*, **1998**. 95(20): p. 11673-11678.
[2] Luecke, H., et al., *Journal of Molecular Biology*, **1999**. 291(4): p. 899-911.
[3] Numbering of the retinal atoms is the same as in Figure 1B.

Table S4. Distance between the key residues and retinal in the last 10 ns simulations of WT-bR$_{cis}$.

Distance/Å	D85-T89	D212-Y185	D212-Y57	R82-D85	D96-T46	W182-C20 [2]	L93-C20 [2]
WT-bR$_{trans}$	3.00±0.45	2.68±0.15	2.67±0.12	8.31±0.53	2.76±0.13	4.13±0.35	3.99±0.30
1X0S [1]	3.1	2.5	2.8	8.3	2.7	4.0	3.6

[1] Nishikawa, T., et al., *Journal of Molecular Biology*, **2005**. 352(2): p. 319-328.
[2] Numbering of the retinal atoms is the same as in Figure 1B.

Table S5. Distance between several key residues and retinal in last 10 ns simulations of WT-bR$_{trans}$ and Y185F-bR$_{trans}$.

Distance/Å	W86-C10 [1]	W189-C2 [1]	D85-N [1]	M145-C16 [1]	W182-C20 [1]	L93-C20 [1]
WT-bR$_{trans}$	4.85±0.24	4.28±0.26	3.19±0.19	4.95±0.34	4.10±0.27	3.86±0.29
Y185F- bR$_{trans}$	4.47±0.20	4.06±0.22	2.79±0.09	4.51±0.34	4.50±0.33	4.05±0.39

[1] Numbering of the retinal atoms is the same as in Figure 1B.

Table S6. Distance between several key residues and retinal in last 10 ns simulations of WT-bR$_{cis}$ and Y185F-bR$_{cis}$.

Distance/Å	Y/F185-N [1]	W189-C2 [1]	W86-C10 [1]	M145-C16 [1]	W182-C20 [1]	L93-C20 [1]
WT-bR$_{cis}$	3.90±0.12	3.99±0.24	4.34±0.26	4.75±0.68	4.13±0.35	5.39±0.30
Y185F- bR$_{cis}$	4.95±0.42	4.43±0.26	4.59±0.24	3.99±0.33	3.90±0.25	5.25±0.29

[1] Numbering of the retinal atoms is the same as in Figure 1B.

Table S7. Distance between several key residues and retinal in last 10 ns simulations of M_1-es state.

Distance/Å	Y/F185-N [1]	W86-C10 [1]	Y57-N [1]	M145-C16 [1]	W182-C20 [1]	L93-C20 [1]
WT-M_1-es	5.28±0.28	4.52±0.24	7.04±0.43	4.13±0.28	4.22±0.27	3.97±0.36
Y185F-M_1-es	5.15±0.34	4.72±0.28	7.45±0.48	4.49±0.36	4.09±0.31	4.17±0.34

[1] Numbering of the retinal atoms is the same as in Figure 1B.

Table S8. Distance between several key residues and retinal in last 10 ns simulations of M_1-cs state.

Distance/Å	W138-C4 [1]	W189-C2 [1]	W86-C10 [1]	M145-C16 [1]	W182-C20 [1]	L93-C20 [1]
WT-M_1-cs	4.67±0.30	4.05±0.23	4.96±0.28	4.86±0.41	3.86±0.24	5.24±0.33
Y185F-M_1-cs	4.31±0.31	3.69±0.25	4.80±0.28	4.50±0.41	3.97±0.26	5.35±0.38

[1] Numbering of the retinal atoms is the same as in Figure 1B.

Table S9. Distance between several key residues and retinal in last 10 ns simulations of M_2 state.

Distance/Å	Y/F185-D212	D212-Y57	Y83-E194	E194-E204
WT-M_2	3.50±0.14	2.70±0.14	2.69±0.13	2.63±0.15
Y185F-M_2	3.95±0.33	2.69±0.15	2.71±0.15	2.62±0.16

[1] Numbering of the retinal atoms is the same as in Figure 1B.

Table S10. Distance between the key residues and retinal in the last 10 ns simulations of M_2' state.

Distance/Å	Y/F185-D212	Y/F185-C13 [1]	W182-C20 [1]	L93-A215	F219-L93	T46-D96
WT-M_2'	3.54±0.14	3.48±0.19	4.00±0.21	4.97±0.56	6.21±0.42	3.56±0.39
Y185F-M_2'	4.11±0.37	4.47±0.29	4.41±0.28	5.14±0.86	7.02±0.28	2.81±0.22

[1] Numbering of the retinal atom is the same as in Figure 1B.

Table S11. Distance between the key residues and retinal throughout the whole simulations of M_2' state.

Distance/Å	Y/F185-D212	Y/F185-C13 [1]	W182-C20 [1]	L93-A215	F219-L93	T46-D96
WT-M_2'	3.48±0.13	3.48±0.19	3.64±0.24	4.68±0.41	5.81±0.58	3.80±0.80
Y185F-M_2'	4.09±0.37	4.37±0.27	4.13±0.32	5.66±0.71	6.87±0.42	3.03±0.67

[1] Numbering of the retinal atom is the same as in Figure 1B.

Video S1. Dynamic changes in the cytoplasmic region residues in WT-M_2'.

Video S1.mp4

Video S2. Dynamic changes in the cytoplasmic region residues in Y185F-M_2'.

Video S2.mp4

Video S3. Dynamic changes in the cytoplasmic region residues in WT-bR_N.

Video S3.mp4

Video S4. Dynamic changes in the cytoplasmic region residues in Y185F-bR_N.

Video S4.mp4
